# Serum periostin levels following small bone fractures, long bone fractures and joint replacements: an observational study

**DOI:** 10.1186/s13223-018-0254-9

**Published:** 2018-07-26

**Authors:** Rachel Varughese, Ruth Semprini, Claire Munro, James Fingleton, Cecile Holweg, Mark Weatherall, Richard Beasley, Irene Braithwaite

**Affiliations:** 1 Medical Research Institute of New Zealand, Private Bag 7902, Newtown, Wellington 6242 New Zealand; 2 Victoria University of Wellington, Wellington, New Zealand; 3 Genentech Inc, San Francisco, USA; 4 University of Otago, Wellington, New Zealand; 5 Capital & Coast District Health Board, Wellington, New Zealand

**Keywords:** Periostin, Bone healing, Asthma, Monoclonal antibody therapy

## Abstract

**Background:**

In asthma, serum periostin may potentially be used as a biomarker in the management of patients with Type-2 eosinophilic airway inflammation. However, serum periostin may be influenced by factors other than Type 2 inflammation, potentially confounding its interpretation. We aimed to measure change in periostin following bone injury.

**Methods:**

102 adults without asthma were recruited into three groups: joint replacement surgery, long bone fracture, short bone fracture. Participants underwent seven measurements of serum periostin over 26 weeks after bone injury, and prior to surgery in the joint replacement group. Differences in periostin were measured using a ratio of geometric mean (RGM), with comparison made with pre-surgery (joint replacement) or 26 week (long and short fracture) reference measurements.

**Results:**

In the joint replacement group, periostin fell within 48 h (RGM 0.80, 95% CI 0.75–0.86), then increased to a maximum at 8 weeks (RGM 1.89, 1.77–2.02) and by 26 weeks remained above the reference measurement (RGM 1.27, 1.19–1.36). In the long bone fracture group, periostin was reduced at 48 h (RGM 0.76, 0.71–0.83) and then progressively increased to a maximum at 8 weeks (RGM 1.15, 1.06–1.23) compared with the reference measurement. In the short bone fracture group, periostin was reduced at 48 h (RGM 0.9, 0.85–0.95) but was not different from after week 1 compared with the reference measurement.

**Conclusions:**

Serum periostin levels are influenced by bone injury. The timing and extent of bone injury needs consideration if periostin is used as a biomarker in the management of eosinophilic asthma.

*Trial registration* This trial was prospectively registered with the Australia New Zealand Trials Registry on Feb 7 2014, (ACTRN12614000151639: https://www.anzctr.org.au/Trial/Registration/TrialReview.aspx?id=363881).

**Electronic supplementary material:**

The online version of this article (10.1186/s13223-018-0254-9) contains supplementary material, which is available to authorized users.

## Background

In asthma, serum periostin may identify patients with Type-2 dependent eosinophilic airway inflammation [1], predict sputum eosinophilia and eosinophilic infiltration of the lung parenchyma [2], and in conjunction with high FeNO identify patients at highest risk of FEV1 decline and asthma exacerbation, regardless of inhaled corticosteroid (ICS) use [3]. Additionally serum periostin may identify patients who respond to monoclonal antibody therapy directed against interleukin (IL)-4 receptor alpha (Rα) [4], IL-13 [5, 6] and immunoglobulin E (IgE) [7]. Serum periostin levels do not need to be adjusted to take account of a patient’s age, sex, or common comorbidities [8], although periostin levels may be lower in smokers [8], in those with elevated BMI [8], and those with pre-extant osteoarthritis [9], and may be raised in a number of cancers [10]. Treatment with inhaled [11] or systemic [12] corticosteroids result in a modest reduction in serum periostin levels. Serum periostin may also be affected by factors other than those related to Type 2 inflammation or its treatment, such as bone injury. This is an important consideration as periostin is a matricellular protein which influences tissue development, remodeling and wound repair in adults [13]. Periostin levels have been shown to be elevated in patients with osteoporotic fracture [14], and in those who have suffered hip fracture [15]. The magnitude of the change in periostin relative to fracture size remains unquantified, as does the period of time in which serum periostin levels return to baseline.

Periostin, originally termed osteoblast-specific factor 2, was first identified in 1993 in mouse osteoblasts [16] and is encoded by the POSTN gene, located on chromosome 13 [17]. Its structure consists of a cysteine-rich domain at its N-terminal, four fasciclin-1 (FAS1) domains in the middle and an alternative splicing domain at its C-terminal [16]. These cysteine-rich domains are likely responsible for the binding of integrins, proteins which regulate cell adhesion and mobility [18], which are key in its role in connective tissue remodeling and repair. In asthma, Type-2 inflammatory cytokines IL-4 and IL-13 induce upregulation of the POSTN gene responsible for the encoding of periostin [19]. Periostin in turn binds to integrins on eosinophils, promoting localisation and adhesion to the airways [19], thereby promoting inflammation and fibrosis [10, 20]. Not only does periostin exist in the basement membrane and mesenchymal tissues of the lung, periostin isomers exist in skeletal muscle, myocardium and heart valves, skin, periodontal ligaments, tendons, neoplastic tissues, and bones.

Periostin is so-named because high concentrations are found within periosteum [21], the layer of connective tissue surrounding bone. Periosteum in adults is composed of two layers: an outer, fibrous layer composed of fibroblasts and mesenchymal cells and an inner, osteogenic layer, which includes mesenchymal stem cells, osteoblasts and endothelial pericytes [22]. Cells in the inner layer are responsive to a wide range of growth factors and proteins and become highly active during bone remodeling, promoting callus formation and osteogenesis. The periosteum plays a central role in the process of fracture repair. Periostin release during bone injury is stimulated by the release of inflammatory cytokines, such as bone morphogenic proteins from the periosteum, which stimulate bone production [23]. In mouse models periostin is important for bone development and maintenance. In POSTN gene knock-out mice, affected animals have a dwarfism phenotype, reduced bone mass, and early periodontal disease [18, 24]. Studies using murine models report that periostin mRNA is upregulated after fractures [25], consistent with the integral role periostin has in bone healing.

The aim of this study was to explore the time course of changes in serum periostin after bone injury of different types and severity. Our hypothesis was that serum periostin would increase following bone injury, with the magnitude and duration of any increase larger with bone injury of greater severity, thereby affecting the specificity of periostin in identifying patient endotypes and the ability to plan asthma treatment regimens.

## Methods

This cohort study recruited 102 patients aged 18–75 years from the emergency department, orthopedic fracture clinic, and pre-surgical assessment clinic at Wellington Regional Hospital, Wellington, New Zealand. Three groups of participants were recruited: those with short bone fractures, long bone fractures, and those scheduled for hip or knee arthroplasty. ‘Short bones’ included carpal, metacarpal, tarsal, metatarsal, or vertebral fractures. ‘Long bones’ included tibia, fibula, femur, humerus, radial, ulnar, or rib fractures. Participants were excluded from the study if they had conditions with the theoretical potential to affect serum periostin levels. These included: a doctor’s diagnosis of asthma, bronchitis or chronic obstructive pulmonary disease (COPD); wheeze or use of respiratory inhalers in the previous 12 months; hospital admission, significant surgery (including dental surgery), bone fracture, or use of systemic corticosteroids, all within the 3 months prior to enrolment. Participants were also not recruited if they were pregnant or breastfeeding.

The study conformed to the standards of the Declaration of Helsinki; the Central Regional Ethics Committee of New Zealand granted ethical clearance (13/NTB/186), and written informed consent was obtained from all participants prior to testing.

Participants attended the Medical Research Institute of New Zealand (MRINZ) outpatient facility over a 26-week period (Additional file 1: Figure S1). Participants with fractures were enrolled within 48 h of their injury and then attended for visits at times of 1, 2, 4, 8, 12 and 26 weeks after the fracture. Participants undergoing joint replacement surgery had an additional visit before the surgical procedure to obtain a sample for pre-operative serum periostin, and then were followed-up with the same visit schedule as the fracture groups.

At enrolment, participants completed a General Health Questionnaire, which was based on questions from the American Thoracic Society (ATS) Division of Lung Diseases-78 Questionnaire (DLD-78) [26] and measurement of their body mass index (BMI). Serum periostin was measured at every study visit, including the enrolment visit. Serum periostin was measured using the clinical trial version of the Elecsys^®^ Periostin Immunoassay (Roche Diagnostics, Penzbery, Germany) described previously [27].

### Sample size

The clinically important difference in serum periostin is not known. The sample size of 34 participants in each sub-group was based on detecting a paired difference in periostin of 0.5 of a standard deviation (80% power, Type I error rate 5%, two-sided), which can be considered a ‘medium’ effect size. For comparison between long and short bone fractures groups, a sample size of 68 (34 in each group) was based on 90% power to detect a 0.8 standard deviation difference, which can be considered a ‘large’ effect size.

### Statistical methods

The distribution of serum periostin was right skewed and therefore logarithmic data transformation was applied to the values for analysis purposes so as to meet normality assumptions of the statistical models. The inverse transformation of a difference in logarithms is equivalent to a ratio of geometric mean (RGM) periostin. Periostin values were plotted against time and a locally weighted scatter plot smoother (LOESS) with 90% confidence limits was used to illustrate the pattern of variation with time. Comparison between patient groups used mixed linear models with the individual participant as a random effect and a spatial exponential covariance matrix, using the time between measurements, to estimate the correlation between repeated measurements. Two sets of comparisons were estimated. Firstly, within group models were used to estimate the change from the reference measurement for each patient group; secondly an interaction between patient group and time was assessed. In the fracture groups, as a pre-fracture serum sample could not be obtained from participants, the initial analysis was performed using the first periostin measurement (taken within 48 h) as the reference measurement. However, with the demonstration that the serum periostin falls within 48 h of bone injury associated with joint replacement surgery, it was apparent that the 48 h periostin levels could not be used as the reference measurement in the fracture groups. For this reason, a post hoc analysis was conducted where within group comparisons were estimated using the 26 week values as the reference measurement.

SAS version 9.4 was used.

## Results

**Fig. 1:**
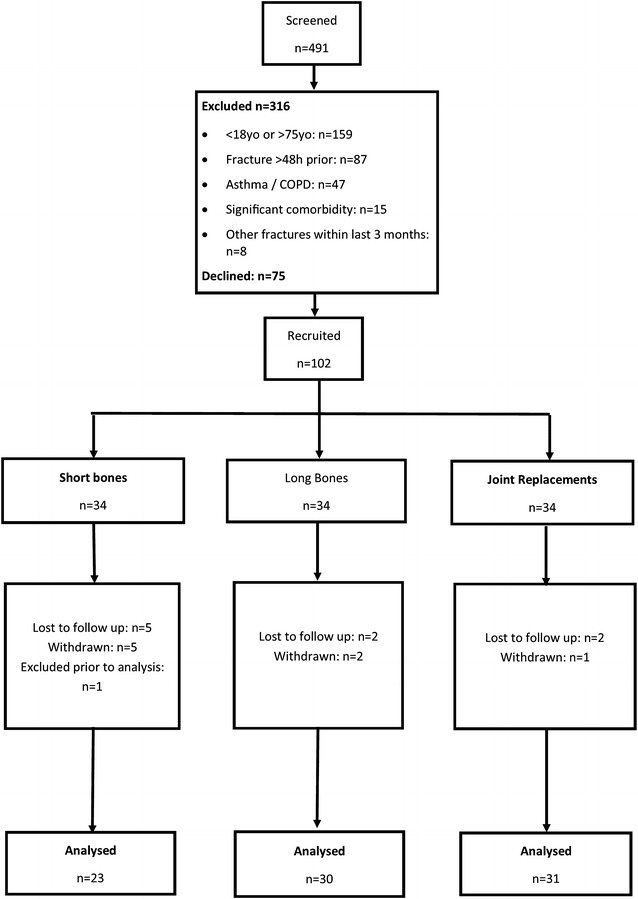
The flow of participants through the study. Participant sustained iliac bone fracture but was allocated to small bone fracture group in error. Therefore excluded from analysis

A total of 102 participants were recruited after 491 people were screened. The flow of participants through the study is shown in Fig. 1. Common reasons for screen failures included being outside of the designated age range, sustaining a fracture more than 48 h prior to screening, declining to participate, and having a doctor’s diagnosis of asthma or COPD. The characteristics of study participants are shown in Table 1. The participants in the joint replacement group were older and had a higher BMI than those in the fracture groups.

**Table 1:** Baseline participant characteristics for all participants

	Joint replacement	Long bone fractures	Short bone fractures
Continuous variables: N = 34			
Age^a^: mean (SD)	60.6 (10.1)	47.9 (17.4)	39.8 (15.7)
BMI^b^: mean (SD)	31.5 (8.3)	26.2 (3.7)	27.4 (4.9)
Categorical variables N/34 (%)			
Female (%)	16 (47)	21 (62)	12 (35)
Atopy^c^ (%)	17 (50)	18 (53)	16 (47)
Non-smoker (%)	31 (91)	29 (85)	27 (79)
Ethnicity			
European (%)	24 (71)	27 (79)	28 (82)
Maori (%)	6 (18)	5 (15)	3 (8)
Pacific (%)	2 (6)	0 (0)	1 (3)
Asian (%)	1 (3)	1 (3)	1 (3)
Other (%)	1 (3)	1 (3)	1 (3)
Steroid medication^d^			
Oral steroids (%)	1 (3)	0 (0)	0 (0)
Intranasal steroids (%)	1 (3)	0 (0)	4 (12)
Topical (%)	0 (0)	0 (0)	1 (3)

*SD* standard deviation, *IQR* interquartile range, *BMI* body mass index, *ng* nanogram, *ml* millilitre

^a^Age: years

^b^BMI: kg/m^2^

^c^Atopy defined as a history of seasonal rhinoconjunctivitis and/or eczema

^d^Participants who took corticosteroid containing medication for any reason during the study period

### Joint replacement group

In this group, there were 18 participants who underwent hip replacement surgery and 16 who had knee replacement surgery, with complete data available on between 31 and 34 participants at each time point. The mean (SD) length of time between the pre-operative reference measurement and the surgery was 46.6 (36.9) days (range 0–170 days). The pre-operative mean (SD) serum periostin was 54.2 (18.0) ng/ml.

**Fig. 2:**
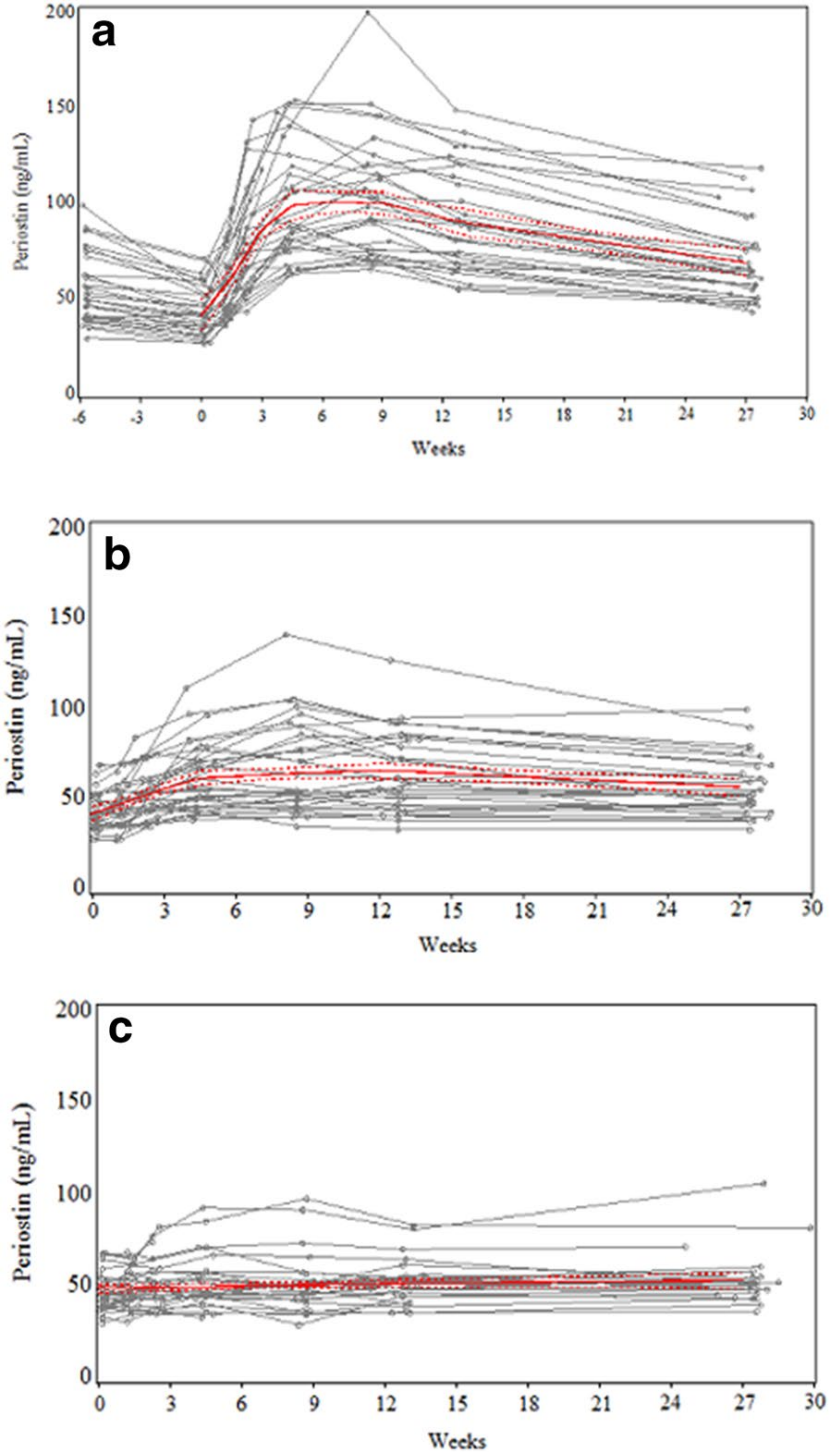
**a** Time course of serum periostin levels over 26 weeks in participants who underwent large joint replacements (including pre-operative reference periostin values). **b** Time course of serum periostin levels over 26 weeks in participants who sustained a long bone fracture. **c** Time course of serum periostin levels over 26 weeks in participants who sustained a small bone fracture. The red solid lines denote the mean and the red dotted lines denote the 90% confidence intervals

Within 48 h of surgery, serum periostin levels fell to a mean (SD) of 43.5 (12.5) ng/ml, represented by a RGM periostin of 0.8, P < 0.001 (Table 2). Serum periostin levels returned to baseline 1 week post-operatively and then progressively increased, with a maximum mean (SD) of 101.3 (31.2) ng/ml, (difference 46.9 ng/ml, RGM periostin 1.89, P < 0.001) at 8 weeks. The periostin level then decreased, but remained above the pre-operative reference level at week 26, when the mean (SD) periostin level was 68.3 (20.7) ng/ml, difference 13.8 ng/ml, RGM periostin 1.27, P < 0.001 (Fig. 2a).

**Table 2:** Serum periostin levels at time points in joint replacement group, and ratio of geometric means compared with pre-operative reference baseline value

Visit	N	Periostin mean (SD)^a^	Change from baseline Mean (SD)^a^	Ratio of geometric means (95% CI)	P
Pre-operative (reference)	34	54.2 (18.0)	–	–	–
Within 48 h	31	43.5 (12.5)	− 11.5 (9.7)	0.80 (0.75–0.86)	< 0.001
Week 1	33	55.5 (17.8)	1.4 (12.4)	1.03 (0.97–1.10)	0.37
Week 2	32	79.0 (27.0)	24.0 (17.3)	1.44 (1.35–1.54)	< 0.001
Week 4	32	97.3 (29.2)	43.6 (1.9)	1.83 (1.72–1.96)	< 0.001
Week 8	31	101.3 (31.2)	46.9 (22.6)	1.89 (1.77–2.02)	< 0.001
Week 12	31	89.8 (26.6)	35.3 (16.3)	1.67 (1.57–1.79)	< 0.001
Week 26	31	68.3 (20.7)	13.8 (12.8)	1.27 (1.19–1.36)	< 0.001

^a^Units: ng/ml

### Long bone fracture group

In this group, 22 had sustained lower limb long bone fractures and 12 sustained upper limb long bone fractures. In 13 participants there were two or more fractures, and there was complete data in 30 participants. The mean (SD) length of time between fracture and the first periostin sample was 1.1 (0.59) days, (range 0–2 days). Given the fall in serum periostin level within 48 h after joint arthroplasty, a post hoc analysis was conducted using the 26 week value as the reference measurement. The mean (SD) periostin at 26 weeks was 56 (16.3) ng/ml.

Compared to the 26 week reference value, the mean (SD) periostin level at 48 h after fracture was lower at 42.7 (10.6) ng/ml, (difference − 13.4 ng/ml, RGM periostin 0.76, P < 0.001) (Table 3). Periostin levels at week two were similar to the 26 week reference values, and then increased progressively to a maximum mean (SD) of 66.6 (25.1) ng/ml at week 8 (difference 11 ng/ml, RGM periostin 1.15, P < 0.001) (Fig. 2b).

**Table 3:** Serum periostin levels at time points in long bone fracture group, and ratio of geometric means compared with week 26 reference level

Visit	N	Periostin mean (SD)^a^	Change from reference Mean (SD)^a^	Ratio of geometric means (95% CI)	P
Within 48 h	34	42.7 (10.6)	− 13.4 (12.4)	0.76 (0.71–0.83)	< 0.001
Week 1	34	45.7 (11.9)	− 11.2 (10.9)	0.81 (0.76–0.88)	< 0.001
Week 2	34	52.5 (13.0)	− 3.7 (8.2)	0.94 (0.87–1.02)	0.12
Week 4	33	62.0 (18.2)	− 5.8 (10.9)	1.10 (1.02–1.18)	0.015
Week 8	34	66.6 (25.1)	− 11.0 (16.1)	1.15 (1.06–1.23)	< 0.001
Week 12	32	63.9 (21.9)	− 7.7 (10.9)	1.11 (1.04–1.20)	0.004
Week 26 (reference)	30	56.0 (16.3)	–	–	–

^a^Units: ng/ml

### Short bone fracture group

Of the 34 participants recruited, 24 had complete data. Within this group: nine had sustained a metacarpal or carpal fracture, five had a metatarsal or tarsal fracture, 16 people had fractured phalanges (11 in the hand, five in the foot), two people had calcaneus fractures and one person fractured their talus. Three participants had sustained two or more fractures. The mean (SD) length of time between fracture and the first periostin sample was 1.29 (0.68) days, (range 0–2 days). The mean (SD) periostin at 26 weeks was 53 (14.8) ng/ml.

Compared with the 26 week reference value, the periostin level at 48 h was lower, with a mean (SD) of 46.1 (10) ng/ml (difference − 5.7 ng/ml, RGM periostin 0.9, P < 0.001) (Table 4). Periostin levels at week two were similar to the 26 week measurements (mean 49.9 ng/ml, difference − 2.4 ng/ml, RGM periostin 0.97, P = 0.37) and did not change significantly after this time point (Fig. 2c).

**Table 4:** Serum periostin levels at time points in short bone fracture group, and ratio of geometric means compared with week 26 reference level

Visit	N	Periostin mean (SD)^a^	Change from reference Mean (SD)^a^	Ratio of geometric means (95% CI)	P
Within 48 h	34	46.1 (10.0)	− 5.7 (14.8)	0.90 (0.85–0.95)	< 0.001
Week 1	32	47.8 (9.6)	− 3.5 (11.2)	0.93 (0.88–0.99)	0.021
Week 2	29	49.9 (12.1)	− 2.4 (7.8)	0.97 (0.93–1.03)	0.37
Week 4	28	51.5 (14.2)	0.5 (7.3)	1.00 (0.95–1.06)	0.87
Week 8	27	50.6 (15.7)	− 1.0 (6.9)	0.97 (0.92–1.02)	0.24
Week 12	27	51.6 (12.1)	− 0.3 (6.9)	1.00 (0.94–1.05)	0.99
Week 26 (reference)	24	53.0 (14.8)	–	–	–

^a^Units: ng/ml

Raw serum periostin values, for all three groups and all visits, are shown in Additional file 1: Table S1a–c.

### Comparison between the groups

The periostin levels were similar between the joint replacement and long fracture groups at 48 h, but the joint replacement group had higher periostin levels at all subsequent time points to week 26 (Table 5).

The periostin levels were similar between the long bone fracture and short bone fracture groups at 48 h to week 2. The long bone fracture group had higher periostin levels at weeks 4, 8 and 16, but there was no difference between the two fracture groups at week 26 (Table 5).

**Table 5:** Change in serum periostin over time: differences between the three groups

Visit	Difference in logarithm periostin (95% CI)	Ratio of geometric mean periostin (95% CI)	P
Joint replacement minus long bone fracture			
Within 48 h	− 0.01 (− 0.14 to 0.12)	0.99 (0.87–1.13)	0.90
Week 1	0.18 (0.05–0.31)	1.20 (1.05–1.37)	0.007
Week 2	0.37 (0.24–0.51)	1.45 (1.27–1.66)	< 0.001
Week 4	0.47 (0.34–0.60)	1.59 (1.40–1.82)	< 0.001
Week 8	0.45 (0.32–0.58)	1.57 (1.37–1.79)	< 0.001
Week 12	0.36 (0.23–0.49)	1.43 (1.25–1.63)	< 0.001
Week 26	0.19 (0.06–0.33)	1.22 (1.06–1.39)	0.004
Long bone fracture minus short bone fracture			
Within 48 h	− 0.09 (− 0.22 to 0.04)	0.92 (0.80–1.04)	0.18
Week 1	− 0.05 (− 0.18 to 0.08)	0.95 (0.84–1.08)	0.45
Week 2	0.06 (− 0.07 to 0.19)	1.06 (0.93–1.21)	0.35
Week 4	0.19 (0.06–0.32)	1.21 (1.06–1.38)	0.005
Week 8	0.28 (0.14–0.41)	1.32 (1.15–1.50)	< 0.001
Week 12	0.21 (0.07–0.34)	1.23 (1.08–1.40)	0.002
Week 26	0.11 (− 0.03 to 0.24)	1.11 (0.97–1.27)	0.11

## Discussion

This study has shown that serum periostin levels are influenced by bone injury and repair. A biphasic response is apparent in which the periostin level fell in the first 48 h of injury, and then progressively increased over the following weeks as the bone repaired. The magnitude and duration of this increase in periostin levels was determined by the severity of the bone injury and associated soft tissue damage, with the periostin level increasing almost two-fold 8 weeks after joint replacement surgery, and still remaining above pre-surgery reference levels at 26 weeks. Additionally, even with small bones, measurement within the first 2 weeks of a fracture may result in a lower than usual periostin level. The clinical significance of these findings is that a history of the timing and extent of recent bone injury needs to be considered if serum periostin is to be used to guide treatment decisions in the management of eosinophilic asthma. The time-course of change in periostin levels in the immediate period after joint replacement surgery and long bone fracture is consistent with that seen in a previous study of periostin levels after hip fracture [15]. Elevated periostin levels with fracture have also been observed in patients with radiological evidence of osteoporotic fracture [14]. Neither of these studies obtained baseline periostin levels prior to fracture, which we were able to do in the joint replacement group.

We did not anticipate that there would be a biphasic pattern with the initial drop of serum periostin within 48 h of the index event, or the long duration of the subsequent progressive increase. However, these findings may be explained by the mechanisms of bone healing. Bone healing is a complex process involving a cascade of events and changes in the expression of several thousand genes. The physiological processes involved in fracture healing occurs in three stages: inflammatory, proliferative and reparative, and remodeling. The inflammatory phase occurs immediately, involving hematoma and granulation tissue formation. It is during this phase where pro-inflammatory molecules such as tumor necrosis factor-α (TNF-α), IL-1 and IL-6, are secreted promoting angiogenesis and proliferation of osteoblasts and osteoclasts [28]. The acute inflammatory response peaks within the first 24 h and is complete after 7 days. The proliferative and reparative phase involves the proliferation and transformation of periosteal cells, production of a cartilaginous callus followed by resorption and replacement with new trabecular bone. During this phase osteogenic molecules, such as bone morphogenetic protein-2 (BMP-2), up-regulate periostin synthesis within the periosteum and the soft callus [23]. Murine models report high concentrations of periostin mRNA within immature osteoblasts in periosteal tissues within the first 3 days of fracture healing [25]. This creates a periostin-rich environment resulting in the proliferation, differentiation and adhesion of osteoblasts [29]. The final process is remodeling, occurring from roughly 6–8 weeks after a fracture, whereby trabecular bone is resorbed by osteoclasts, and then compact bone is deposited within the resorption pit. During this phase, in mouse models, the periosteum’s metabolic activity slows [25], resulting in a fall in periostin.

It is possible that the immediate fall in serum periostin is due to the ‘mopping up’ of periostin as part of the inflammatory response. Alternatively, early periostin isoforms secreted by the periosteum may exert paracrine effects and not be measurable in serum, similar to that observed in cutaneous wound repair [30], resulting in lower serum periostin values. The later rise in serum periostin may be due to ongoing up-regulation of periostin synthesis, through the proliferative and reparative phases, resulting in excess production of periostin and its release into the circulation. These phases last for 6–8 weeks, consistent with the time course of the peak serum periostin in the joint arthroplasty and long bone fracture groups occurring 8 weeks after bone injury, after which the levels gradually reduced during the final remodeling phase. The widespread use of non-steroidal anti-inflammatory (NSAIDs) analgesics intra- and post-operatively, and also after the fractures, may have contributed to the patterns observed, with 60% of our patients using NSAIDs at some point during the study. Through inhibition of prostaglandins, NSAIDs impair or delay bone healing and decrease the integrity of the healing bone [31], an effect which may contribute to the slow and progressive rise in periostin levels.

There are a number of methodological issues that are relevant to the interpretation of the study findings. Three bone injury groups were studied to enable differing types and magnitudes of bone and associated soft tissue injury to be examined. The contribution of soft tissue injury to the changes in periostin levels may have been significant, as periostin is also secreted by tissue fibroblasts in response to injury [13]. Whether the bone was weight bearing was also likely to be a contributing factor, as mechanical stress in weight bearing bones is associated with periostin levels [32]. Although 16 participants fractured more than one bone, each participant was allocated to the group depending on the largest bone fractured, under the assumption that this would have the greatest influence on serum periostin values. While a number of other conditions such as atopic dermatitis [33], rhinosinusitis [34, 35], and malignancies [10, 36] may contribute to elevated periostin levels we did not utilize these as exclusion criteria. As this was a study of change in serum periostin after fracture, we considered that inclusion of patients with these conditions would not have influenced the findings. Serum periostin levels are lower in those with higher BMIs [8]. We analysed the mean change in periostin levels within each group after fracture and each patient was followed over time, serving as their own control. Therefore the results of the study are unlikely to be affected by variation in BMI. Periostin has been shown to be higher in children, likely due to bone growth [37], therefore we only recruited adults in whom we have shown age does not influence periostin levels [8]. Our decision not to exclude acute brain injuries, also known to contribute to elevate periostin levels [38], is likely to improve the generalizability of our findings.

Joint replacement surgery, which was chosen as the model of the greatest magnitude of bone and soft tissue damage, also provided the advantage of allowing a pre-injury periostin measurement to be obtained as a reference baseline. As it was not possible to obtain a pre-fracture sample from those who had sustained a fracture, the original intention was to use the 48 h periostin level as the reference baseline for the fracture groups, on the assumption that the periostin level would not have changed from pre-injury reference levels so soon after an injury. However, the 25% reduction in periostin level at this time point in the joint replacement group indicated that this may not be the case. It was for this reason that a post hoc decision was made to use the 26 week value as the reference measurement in both fracture groups.

This study recruited non-asthmatic participants, to minimize confounding of Type 2 inflammation on serum periostin levels if there were exacerbations during the study period, the treatment of which is known to influence periostin levels [12]. However, these results should be generalisable to asthmatics as there are no systematic differences in serum periostin between asthmatic and non-asthmatic adults [11]. Although inhaled and systemic steroids may have a modest short term effect on serum periostin levels, they were used in only 5 and 1% of participants respectively, and as long term treatment, and so this treatment is unlikely to have influenced the results.

## Conclusions

In summary, serum periostin was influenced by bone and soft tissue injury, with a biphasic response, characterized by an initial fall in periostin levels in the first 1–2 weeks, followed by a progressive increase in serum periostin which peaked at 8 weeks, and in the case of bone and soft tissue injury associated with joint replacement surgery, was present for at least 26 weeks. These findings are clinically relevant if periostin is used in the future to guide management and treatment decisions such as identifying patients with eosinophilic airways inflammation, those at risk of FEV1 decline and/or increased asthma exacerbation risk, or who might most benefit from treatment with monoclonal antibody therapy directed against Type 2-related pathways including IL-4Rα, IL-13 and IgE in asthma.

## Additional file

**Additional file 1.** Figure S1, Table S1a–S1c.

## Abbreviations

ATS: American Thoracic Society
BMI: Body mass index
BMP: Bone morphogenetic protein
COPD: Chronic obstructive respiratory disease
IG: Immunoglobulin
IL: Interleukin
MRINZ: Medical Research Institute of New Zealand
mRNA: Messenger ribonucleic acid
NSAID: Non steroidal anti inflammatory drug
SD: Standard deviation
TNF: Tumor necrosis factor
