# Supplementary material for: Serum periostin levels following small bone fractures, long bone fractures and joint replacements: an observational study
Source: Allergy Asthma Clin Immunol. 2018 Jul 26;14:30. doi: 10.1186/s13223-018-0254-9 (PMC6060508; doi:10.1186/s13223-018-0254-9)
Supplement: Supplementary file 1 — Additional file 1. Figure S1, Table S1a–S1c. [file 13223_2018_254_MOESM1_ESM.doc]

**Serum periostin levels following small bone fractures, long bone fractures and joint replacements**

**Online Supplement**

^1^Rachel Varughese BM BCh, ^1,2^Ruth Semprini MB BS BSc, ^1,5^Claire Munro MBChB, ^1,5^James Fingleton PhD, ^3^Cecile Holweg PhD, ^1,4^Mark Weatherall MBChB, ^1,2,5^Richard Beasley DSc, ^1,2,5^Irene Braithwaite MBChB

^1^Medical Research Institute of New Zealand, New Zealand

^2^Victoria University of Wellington, New Zealand

^3^Genentech Inc, San Francisco, USA

^4^University of Otago, Wellington, New Zealand

^5^Capital & Coast District Health Board, Wellington, New Zealand

**Funding**: Genentech Inc, USA

**Correspondence:**

Dr Irene Braithwaite

Medical Research Institute of New Zealand

Private Bag 7902, Newtown

Wellington, 6242, New Zealand

Email: [irene.braithwaite@mrinz.ac.nz](mailto:irene.braithwaite@mrinz.ac.nz)

Telephone: +64-4-805-0245

Fax: +64-4-389-5707

Figure S1. Study visit plan

| Visit No. | Pre-operative^*^ | 1^$^ | 2 | 3 | 4 | 5 | 6 | 7 |
| --- | --- | --- | --- | --- | --- | --- | --- | --- |
| Time Point | 0 | <48hours | 1 week  +/- 3 days | 2 weeks  +/- 3 days | 4 weeks  +/- 3 days | 8 weeks  +/- 3 days | 12 weeks +/- 3 days | 26 weeks +/- 3 days |
| Written informed consent | X | X |  |  |  |  |  |  |
| Enrolment | X | X |  |  |  |  |  |  |
| Measurement of BMI | X | X |  |  |  |  |  |  |
| General Health Questionnaire | X | X |  |  |  |  |  |  |
| Periostin | X | X | X | X | X | X | X | X |

^*^Only applicable to joint replacement group

^$^Study procedures carried out at Visit 1 for long and short bone fracture groups

Table S1a. Raw serum periostin (ng/ml) values for Joint Replacement Group

|  | Visit number | | | | | | | |
| --- | --- | --- | --- | --- | --- | --- | --- | --- |
|  | **Pre-operative (N=34)** | **1 (N=31)** | **2 (N=33)** | **3 (N=32)** | **4 (N=32)** | **5 (N=31)** | **6 (N=31)** | **7 (N=31)** |
| 1 | 77.24 | 62.75 | 96.42 | 143.2 | 153.5 | 145.5 | 136.6 | 92.17 |
| 2 | 85.09 | 68.58 | 54.37 | 81.39 | 134.5 | 199.8 | 148.4 | 113 |
| 3 | 49.13 | 39.58 | 36.37 | 56.98 | 86.27 | 114.9 | 91.22 | 61.52 |
| 4 | 61.97 | 59.16 | 81.57 | 131.6 | 139.9 | 124.9 | 109.3 | 77.95 |
| 5 | 49.14 | 37.53 | 44.52 | 66.45 | 74.1 | 79.48 | 69.05 | 56.97 |
| 6 | 39.26 |  | 36.21 | 65.69 | 65.42 | 67.17 | 64.79 | 45.19 |
| 7 | 45.19 |  | 39.12 | 54.11 | 80.37 | 90.39 | 79.68 | 64.32 |
| 8 | 48.46 | 40.39 | 50.41 | 91.55 | 106.9 | 102.6 | 79.9 | 61.54 |
| 9 | 35.41 | 27.47 | 41.65 | 42.67 | 62.23 | 64.81 | 55.16 | 47.76 |
| 10 | 52.71 | 45.66 | 64.99 | 78.15 | 86.98 | 75.61 | 72.55 | 49.77 |
| 11 | 46.55 | 32.78 | 38.2 | 65.39 | 74.71 | 90.9 | 80.63 | 65.54 |
| 12 | 74.48 | 59.88 | 83.1 | 111.9 | 147.2 | 118.5 | 123.9 | 93.05 |
| 13 | 38.55 | 42.5 | 42.75 | 55.07 | 80.94 | 69.45 | 68.41 | 56.28 |
| 14 | 55.69 | 40.7 | 45.36 | 73.81 | 76.4 | 73.64 | 64.97 | 64.13 |
| 15 | 39.57 | 32.78 | 38.42 | 51.79 | 66.87 | 72.51 | 64.78 | 51.95 |
| 16 | 61.4 | 41.1 | 47.33 | 71.31 | 108.1 | 133.8 | 120.8 | 106.6 |
| 17 | 37.44 | 30.66 | 44.91 | 52.65 | 77.63 | 69.28 | 63.09 | 46.89 |
| 18 | 45.32 | 36.27 | 65.79 | 100.9 | 118.9 | 102.6 | 100.5 | 63.65 |
| 19 | 71.47 | 55.06 | 86.19 | 117.5 |  |  |  |  |
| 20 | 39.26 | 40.75 | 53.91 | 55.21 | 90.74 | 107.3 | 86.27 | 54.3 |
| 21 | 34.01 | 25.78 | 39.02 | 52.55 | 78.79 | 88.98 | 67.36 | 42.72 |
| 22 | 37.15 | 29.3 | 45.83 | 73.71 | 114.5 | 101.4 | 89.55 | 60.25 |
| 23 | 51.08 | 44.94 | 71.75 | 93.62 | 105.7 | 112.1 | 93.43 | 71.09 |
| 24 | 60.01 | 46.93 | 67.05 | 79.14 | 103 | 120.5 | 113.6 | 74.63 |
| 25 | 28.84 | 26.41 | 42.16 |  | 65.06 |  |  |  |
| 26 | 56.27 | 51.26 | 57.18 | 72.6 | 83.97 | 91.54 | 88.22 | 75.96 |
| 27 | 85.66 | 57.95 | 76.45 | 107.5 | 151.7 | 151.4 | 129.4 | 117.8 |
| 28 | 86.83 | 70.28 | 78.49 | 104.8 | 150.5 | 145.1 | 129.7 | 102.7 |
| 29 | 53.57 |  |  |  |  |  |  |  |
| 30 | 37.58 | 34.83 | 35.58 | 48.92 | 63.57 | 70.48 | 56.43 | 49.99 |
| 31 | 41.05 | 29.66 | 40.75 | 53.34 | 62.43 | 70 | 54.23 | 45.74 |
| 32 | 41 | 34.8 | 45.25 | 60.28 | 87.17 | 97.71 | 87.5 | 68.15 |
| 33 | 76.91 | 51.32 | 57.88 | 86.39 | 89.64 | 74.55 | 73.74 | 58.75 |
| 34 | 98.3 | 50.78 | 84.05 | 127.9 | 124.8 | 113.6 | 119.8 | 75.61 |

Table S1b. Raw serum periostin (ng/ml) values for Long Bone Fracture Group

|  |  | Visit number | | | | | |
| --- | --- | --- | --- | --- | --- | --- | --- |
|  | **1 (N=34)** | **2 (N=34)** | **3 (N=34)** | **4 (N=33)** | **5 (N=34)** | **6 (N=32)** | **7 (N=30)** |
| 1 | 44.78 | 51.26 | 63.05 | 68.79 | 84.41 | 82.62 | 68.28 |
| 2 | 50.47 | 57.12 | 60.8 | 67.92 | 63.33 | 61.25 | 63.69 |
| 3 | 51.44 | 54.24 | 53.18 | 54.66 | 53.35 | 54.23 | 54.78 |
| 4 | 32.94 | 43.08 | 49.47 | 57.27 | 51.39 | 55.32 |  |
| 5 | 33.68 | 53.57 | 52.71 | 53.91 |  |  |  |
| 6 | 37.99 | 35.1 | 36.62 | 40.15 | 39.59 | 40.34 | 46.13 |
| 7 | 68.4 | 69.26 | 74.49 | 70.6 | 76.12 | 84.37 | 73.15 |
| 8 | 52 | 56.91 | 59.2 | 66.06 | 76.74 | 85.61 | 67.29 |
| 9 | 34.56 | 36.52 | 45.81 | 51.22 |  | 50.71 | 42.28 |
| 10 | 39.93 | 45.29 | 44.43 | 44.73 | 44.96 | 45.41 | 41.3 |
| 11 | 38.73 | 43.72 | 53.97 | 69.46 | 76.29 | 71.99 | 59.18 |
| 12 | 31.74 | 34.94 | 44.57 | 43.22 | 34.28 | 32.74 | 32.4 |
| 13 | 29.43 | 28.43 | 36.54 | 41.12 | 42.53 | 39.76 | 37.9 |
| 14 | 34.56 | 35.12 | 34.81 | 37.77 | 39.44 | 40.43 | 39.24 |
| 15 | 33.37 | 34.8 | 42.81 | 49.26 | 41.61 | 37.45 | 36.91 |
| 16 | 42.37 | 41.27 | 57 | 78.04 | 100.7 | 91.69 | 76.68 |
| 17 | 42.38 | 49.14 | 60.84 | 70.63 | 96.54 | 78.52 | 60.92 |
| 18 | 27.33 | 27.12 | 33.86 | 44.71 | 48.46 | 57.08 | 50.46 |
| 19 | 41.69 | 46.77 | 49.18 | 49.45 | 45.42 | 54.47 | 51.24 |
| 20 | 48.5 | 48.76 | 52.35 | 55.43 | 52.78 | 49.59 | 52.62 |
| 21 | 37.39 | 40.49 | 42.39 | 42.15 | 44.41 | 43.3 | 47.36 |
| 22 | 68.09 | 68.69 | 72.05 | 76.73 | 70.3 | 81.79 |  |
| 23 | 35.9 | 34.57 | 35.81 |  | 52.57 | 61.18 | 38.63 |
| 24 | 47.57 | 46.76 | 52.95 | 60.48 | 54.41 | 52.19 | 51.6 |
| 25 | 63.56 | 69.31 | 70.54 | 96 | 104.9 | 91.43 | 78.77 |
| 26 | 39.68 | 50.34 | 72.77 | 110.8 | 140.5 | 126.2 | 89.06 |
| 27 | 41 | 40.77 | 57.79 | 79.09 | 70.1 | 58.43 | 58.93 |
| 28 | 47.92 | 39.8 | 50.55 | 60.54 | 85.2 | 70.92 | 47.81 |
| 29 | 27.75 | 26.34 | 43.02 | 82.45 | 91.59 | 71.67 | 45.8 |
| 30 | 40.81 | 41.56 | 43.6 | 49.9 | 52.36 | 42.56 | 46.95 |
| 31 | 34.58 | 33.79 | 39.72 | 45.15 | 49.4 | 46.36 | 46.34 |
| 32 | 41.47 | 44.59 | 43.56 | 49.57 | 54.26 |  |  |
| 33 | 52.41 | 59.8 | 72.3 | 81.33 | 89.65 | 94.17 | 98.97 |
| 34 | 57.71 | 64.86 | 83.4 | 96.72 | 103.8 | 92 | 74.5 |

Table S1c. Raw periostin (ng/ml) values for Short Bone Fracture Group

|  |  | Visit number | | | | | |
| --- | --- | --- | --- | --- | --- | --- | --- |
|  | **1 (N=33)** | **2 (N=31)** | **3 (N=28)** | **4 (N=27)** | **5 (N=26)** | **6 (N=26)** | **7 (N=25)** |
| 1 | 58.94 | 61.29 | 58.91 | 66.46 | 65.58 | 64.18 | 50.41 |
| 2 | 34.34 | 36.36 | 37.13 | 38.74 | 36.1 | 35.18 | 35.79 |
| 3 | 51.49 | 56.86 | 57.06 | 57.26 | 56.94 | 52.26 | 59.96 |
| 4 | 40.4 |  |  |  |  |  |  |
| 5 | 46 | 46.81 | 50.31 | 57.7 | 45.8 | 48.75 | 45.48 |
| 6 | 64.32 | 61.75 |  | 70.61 | 56.46 | 53.77 | 54.68 |
| 7 | 37.98 | 42.97 | 42.43 | 45.26 | 52.35 | 53.72 | 57.77 |
| 8 | 47.56 |  |  |  |  |  |  |
| 9 | 43 | 41.55 | 46.6 | 46.72 | 49.81 | 52.31 |  |
| 10 | 55.42 | 52.57 | 52.4 | 53.39 | 50.48 | 55.73 | 51.99 |
| 11 | 42.5 | 42.09 | 42.89 | 45.24 | 47.27 | 39.94 | 43.59 |
| 12 | 37.4 | 45.3 | 49.99 | 45.89 | 50.8 | 53.23 | 49.87 |
| 13 | 29.43 | 33.92 | 34.58 | 34.43 | 35.69 | 35.04 | 39.29 |
| 14 | 44.52 | 44.64 | 47.61 | 54.64 | 48.93 | 50.53 | 47.92 |
| 15 | 32.46 | 30.11 | 36.11 | 32.62 |  |  |  |
| 16 | 38.93 | 38.43 | 35.13 | 40.17 | 28.58 | 43.99 | 43.17 |
| 17 | 66.79 | 67.77 | 64.35 | 70.55 | 72.9 | 69.74 | 70.98 |
| 18 | 50.46 | 48.94 | 42.85 | 42.46 | 45.5 | 44.75 |  |
| 19 | 55.51 | 50.79 | 51.52 | 49.07 | 50.67 | 51.48 |  |
| 20 | 67.35 | 65.26 | 73.51 |  |  |  |  |
| 21 | 48.4 | 44.71 |  |  |  |  |  |
| 22 | 37.5 | 43.84 | 53.31 |  |  |  |  |
| 23 | 42.09 | 46.78 | 41.39 | 47.23 | 40.27 | 38.33 | 42.77 |
| 24 | 38.29 | 40.84 | 48 | 44.55 | 45.51 | 45.23 | 42.62 |
| 25 | 67.2 | 64.15 | 81.87 | 84.82 | 97.02 | 82.74 | 81.15 |
| 26 | 37.68 | 35.91 | 35.98 | 35.51 | 33.95 | 35.61 |  |
| 27 | 52.1 | 50.42 | 57.72 | 56.09 | 52.43 | 61.17 | 54.1 |
| 28 | 40.64 | 42.48 |  |  |  |  |  |
| 29 | 45.28 | 45.31 | 43.05 | 43.88 | 42.07 | 47.83 | 47.81 |
| 30 | 53.42 | 52.94 | 47.11 | 44.57 | 44.93 | 48.14 | 50.1 |
| 31 | 37.76 | 38.68 | 38.69 | 44.87 | 35.11 | 40.92 |  |
| 32 | 45.31 | 47.89 | 49.39 | 49.4 | 49.51 | 55.39 | 47.71 |
| 33 | 44.52 | 45.04 | 44.41 | 45.77 | 44.49 | 51.77 | 52.34 |
